# How do group workers respond to suicidal behavior? Experiences and perceptions of suicidal female adolescents residing in secure residential youth care in the Netherlands

**DOI:** 10.1371/journal.pone.0283744

**Published:** 2023-03-30

**Authors:** S. P. T. Kaijadoe, H. Klip, A. de Weerd, E. A. van Arragon, K. S. Nijhof, A. Popma, R. H. J. Scholte

**Affiliations:** 1 Karakter Child and Adolescent Psychiatry University Centre, Nijmegen, The Netherlands; 2 Experienced Expert, Student Psychology, Nijmegen, The Netherlands; 3 Pluryn, Nijmegen, The Netherlands; 4 Radboud University Nijmegen, Nijmegen, The Netherlands; 5 Vrije Universiteit Amsterdam, Amsterdam, The Netherlands; Pontificia Universidad Católica de Chile: Pontificia Universidad Catolica de Chile, CHILE

## Abstract

**Background:**

Adolescent suicidal behavior, including non-suicidal self-injury, is increasingly prevalent in Secure Residential Youth Care (SRYC) in the Netherlands. Group workers play a vital role in the well-being and functioning of adolescents in SRYC as they interact with adolescents on a daily basis. However, we have little understanding of how adolescents perceive group workers’ responses to suicidal behavior and we lack knowledge about the impact of these responses on adolescents and the group climate.

**Aim:**

The aim of this study is to explore (a) how adolescents value group workers responses towards suicidal behavior and (b) the impact of these responses on adolescents, as well as (c) on the group climate. The results can be used to develop care-policy to improve care for suicidal adolescents in SYRC.

**Method:**

Eleven suicidal female adolescents residing in SRYC were interviewed. All adolescents had previously displayed suicidal behavior, including non-suicidal self-injury. Interviews were analyzed using grounded theory.

**Conclusion:**

This study presents the perceptions of suicidal female adolescents residing in SRYC about group workers’ responses on suicidal behavior. Adolescents prefer group workers who react responsive to suicidal behavior. Responsive care, trust and connectedness help adolescents disclose their suicidal thoughts. Participants criticize group workers who are non-responsive as being distant, and their relationship with these group workers lacked trust, communication, a sense of connection, or personal depth. All adolescents underline the devastating impact of involuntary seclusion, and stress the importance of being able to disclose without fear of coercive consequences. Findings indicate that non-responsive reactions contribute to an increase in suicidal distress as well as a closed group climate.

## Introduction

In the Netherlands, suicide is the main cause of death amongst young people aged 10 to 20 years [1]. Comparable to suicide numbers worldwide [2], the suicide rate among young people in the Netherlands has increased dramatically during recent decades. In 1970 the number of completed suicides among young people was approximately 1.6 suicides per 100 thousand, whereas this number has currently almost doubled to 3.1 suicides per 100 thousand [3]. Nowadays, on average every week one adolescent commits suicide in the Netherlands [3]. Even though the absolute numbers seems low, suicide has a significant social impact, and the loss of a loved one by suicide has lasting and far-reaching consequences for families, friends, care providers and communities long after the tragedy has taken place [4, 5].

Suicide and suicidal behavior among adolescents is influenced by multiple, interacting risk factors, [6, 7]. They include child, environmental and family-related factors. The child factors consist of internalizing psychiatric problems such as a tendency to internalize, being emotionally withdrawn, having moderate emotional skills, low self-esteem, limited frustration tolerance, strong ‘black and white’ thinking, the idea of total failure and serious questions of identity [8]. In the Netherlands, adolescents with internalizing psychiatric are increasingly referred to Secure Residential Youth Care (henceforward referred to as SRYC) instead of being referred to mental health care as was the case in the past [9]. In 2015 a large decentralization took place in youth care with whom the Dutch governments aimed to transform the youth care system. The intention of this reform was to simplify the system of youth care and to make it more effective and efficient. Moreover, it intended to strengthen the resilience of youngsters and their families. Given the high costs associated with placement in residential care, the transition of 2015 entailed budget cuts as well [10]. As a result of these budget cuts, youth mental health care clinics had to reduce their number of beds and young people with internalizing psychiatric problems are increasingly referred to SRYC facilities [9]. Internalizing psychiatric problems include depression, anxiety disorders and psychosomatic complaints which are prevalent among an estimated 45–78% of adolescents in SRYC [11]. Moreover, one third of the adolescents with internalizing psychiatric problems have suicidal ideations or are involved in self-harm [12]. This increase of adolescents with internalizing psychiatric problems is problematic as SRYC is mainly developed for externalizing problems [12]. There is a high prevalence of many risk factors in youth residing in SRYC [12]. Hence, it is not surprising that adolescents residing in SRYC are also at a higher risk for suicide and suicidal behavior.

In 2020 more than 400,000 (12%) young people up to the age of 18 received some form of youth care of which approximately 40,000 were placed in some form of residential care (including foster care, family homes). Of these, 2000 adolescents were treated in SRYC after a decision by the juvenile court. On average, they resided 6,5 months in SRYC [13]. Adolescents placed in SRYC show severe mental and behavioral health needs [14] and are a danger to themselves or their environment [15, 16]. SRYC is the most restrictive and expensive type of residential care, and is often seen as a “last resort” of youth mental health care intervention [17–19]. In SRYC, approximately 8 to 12 adolescents live together in one group, although nationally we do see a transformation to smaller groups of 4 to 6 adolescents [20, 21]. Treatment in SRYC consists of therapy activities in the living group, combined with individual therapies, counselling and pharmacotherapy which are offered on site. Staff in teams are multi-disciplinary and generally led by psychologists or educational scientists, and/or psychiatrists. Treatment in SRYC can differ by various factors. These factors include treatment organization, staff qualification, family involvement, staff-adolescent ratio, as well as support after being discharged [22]. Adolescents in SRYC may encounter restricted freedom of movement (locked doors), limited communication (phone use or visits) and control measures such as urine control and examination of the body and clothes. Moreover, coercive measures such as restraint or seclusion can be used in case of incidents (e.g. suicidal threat, self-harm, aggressive behavior, rule violation) [23, 24]. Next to school attendance (which is offered on site), adolescents spend most of their time within the living group environment where they receive care and support 24 hours a day by trained group workers [25]. Generally, group workers in SRYC are trained social workers with vocational education or higher professional education. The current study explores how adolescents in SRYC experience the way group workers react and respond towards suicidal behavior.

The ability of professionals to establish good, responsive relationships with residents is an important factor in enhancing treatment motivation in order to achieve positive treatment outcomes [26–28]. Treatment motivation in SRYC is seen as a key to success for effective treatment [29, 30] but often SRYC residents suffer from low levels of treatment motivation [31]. This lack of treatment motivation may arise from their mental health problems, a long history of ineffective treatments, or may be a result of enforced admission into residential care [32, 33]. Moreover, group workers should provide for positive, responsive relationships with adolescents in care, in order to establish a positive group climate [34]. A positive group climate consists of a living environment with support and structure in which growth and a normal development of residents is stimulated and opportunities for learning are created, whilst responsiveness and clear (life) rules are provided for [32, 34, 35]. Responsive reactions refer to the manner in which a group worker responds to signals indicating the adolescent is unwell and in need of emotional support in order to cope with problems and stress. In sum, group workers play a vital role in the well-being and functioning of adolescents residing in SRYC [36].

The increase of suicidal adolescents and completed suicides in SRYC [9, 37] challenges group workers in their daily task as the care provided for in SRYC is traditionally aimed at teenagers with externalizing behavior (i.e., lying, no respect for others, verbal or physical aggression) [12]. The experience of a completed suicide can negatively affect both professional and personal lives and the most usual emotional reactions reported by professionals include guilt and self-blame, anger, shock, fear, concern, loss of self-confidence, and feelings of incompetence or failure [38–40]. Moreover, professionals in SRYC indicate that they are not sufficiently trained to deal with adolescent suicidal behavior and professionals feel insecure [41, 42], causing them to act more controlling towards suicidal adolescents [43] than necessary [35]. The use of coercive measures (for example fixating or isolating juveniles) may endanger the therapeutic relationship [44–46] which is alarming as one of the important features in the treatment of suicidal adolescents is to provide for positive, responsive relationships with adolescents [47]. Having a bond of trust enables adolescents to talk about their suicidal feelings, which contrary to popular belief, reduces suicidal feelings, rather than increasing them [48]. Nonetheless, professionals are often reluctant to discuss the suicidal feelings of adolescents and find it hard to ask “the question” about suicidal ideation [49]. Research shows that health care professionals often lack specialized interventions such as suicide prevention when working with suicidal patients [50, 51]. Moreover, professionals indicate that working with suicidal adolescents can be very intrusive and the majority experiences difficulties in identifying with the suicidal distress of adolescents, which limits their capacity to understand, listen and empathize [52]. As consequence, the adolescent does not feel understood, which may increase the risks for incidents as a body of research shows that the experience of incomprehension and of feeling unheard is central to the suicidal process of suicidal adolescents [53–56]. Therefore, it is important to understand from an adolescent perspective how professionals in SRYC address the needs of suicidal adolescents.

The self-determination theory (hereafter referred to as ‘SDT’) describes the importance of three basic psychological needs of suicidal adolescents [57]. Hence from a SDT stance, a starting point to achieve enduring behavior changes with youth in residential treatment is the focus on youths’ individual needs and intrinsic motivations [29, 58, 59]. A basic principle of the SDT is that social environments supporting the three basic psychological needs for autonomy, relatedness and competence are important for psychological health, treatment motivation and well-being. The need for autonomy refers to the need to self-regulate one’s experiences and actions and experiencing a sense of choice or psychological freedom [60]. The need for relatedness concerns the desire to feel socially connected [61] and the need for competence refers to the ability to affect the environment and to attain desired outcomes within it [62]. A variety of studies show that a lack of these three basic psychological needs may lead to an increased suicide risk in adolescents [57, 63]. Suicidal adolescents urge the need to be understood by those surrounding them (including empathetic professionals) in order to recover from suicidality [64]. Unconditional attention and support [65, 66] of the health care provider is an important asset in treatment, which corresponds to the need for relatedness. Feeling trapped in suicidal thoughts, and a narrowed vision is characteristic for people with suicidal behavior [67]. Entrapment is expressed in hopelessness and not knowing how to proceed in live (lack of competence). The experience of shame [64] and failure [53, 54, 68], mental pain [55, 65] and despair [69] is at the heart of this distress. The fear of being judged by others amplifies the feeling of solitude and isolation [55] (lack of relatedness). Many suicidal youth have the impression that they have lost control of their existence [70], and dealing with problems of strong emotions seems impossible to them [66]. However, by talking about themselves, by understanding themselves suicidal adolescent can regain control over their lives [66] and meet the need for autonomy and competence [62, 71, 72]. Within the living group environment, group workers are very important in fulfilling these needs as they interact with residents on a daily basis [36, 73, 74].

Though research has been conducted on group climate in living groups in SRYC [75, 76], we do not know what the impact is of group workers responses to suicidal behavior on the group climate. The latter is important for it is now well established from a variety of studies that group climate plays a critical role in residential youth care [77–79]. Research of De Valk [43] points out that in a repressive closed group climate the use of controlling measures increases. This may lead to an increase in stress among adolescents and a reduction of connectedness, competence and autonomy, the three basic psychological needs according to the self-determination theory [62, 80]. Ultimately, this can increase suicidal behavior [35] as no contact with a suicidal adolescent increases the risk of suicidal incidents [47]. Therefore, increasing our knowledge on the impact of group workers responses on the group climate can play an important role in addressing suicide prevention and quality of care for suicidal adolescents in SRYC.

The goal of the present study is to explore how adolescents in SRYC experience the way group workers respond towards suicidal behavior. Pedagogical interventions of group workers have a huge influence on the quality of care for adolescents in SRYC [25]. Group workers interact with adolescents on a daily basis and as such they are an important factor for the well-being and functioning of adolescents [74]. Moreover, as described earlier, professionals in SRYC point out that they are insufficiently trained to meet the needs of suicidal adolescents [42]. Therefore it is important to know how adolescents perceive the responses of group workers to suicidal behavior. However, research on this topic is lacking as most studies on SRYC concern effect studies [81] and only a few studies report on residents’ perceptions on the treatment they receive [82, 83].

Although there is no literature from the perspective of adolescents on group workers’ responses to suicidal behavior, comparable research on the topic of self-harm is available. Johnson and colleagues (2017) asked adolescents showing self-harm about effective support and counterproductive behavior of professionals [84]. The young people noted numerous responses that had both helpful and unhelpful effects, such as increased observation, removal of means and extra collaborative support. O’Brien et al. (2019) focused on the factors that facilitated the process from suicide ideations to a suicide attempt, but did not focus on the perception of adolescents on the care they received [85]. The work of Grimmond et al. (2019) provides an excellent overview of qualitative studies on the experiences of suicidal people 25 years and younger but their review did not entail the view of adolescents residing in SRYC [86]. The latter also applies to the work of Engström and colleagues (2020) who explored adolescents’ experiences of staff’s different interaction styles in SRYC. Notwithstanding, their research did not aim specific towards the care for suicidal young people [82].

Therefore, this qualitative study explores how adolescents in SRYC experience group workers responses to suicidal behavior. We also explore the impact of these responses on adolescents’ well-being and functioning as well as on the group climate. The results can be used for the development of care-policy in practice in order to improve the care for suicidal adolescents. To note, in this study the term “suicide” is referred to when the victim has committed an act with the aim of taking his own life. We use the term "suicidal behavior" to refer to thoughts, preparatory acts and attempts that express a certain intention to kill oneself (including NSSI, regardless its intention).We use the term “suicidal distress” to refer to a crisis situation in which the person in crisis is suicidal.

## Method

### Study design

A qualitative approach was used to enable an inside perspective and a deeper understanding of the experiences of adolescents living in SRYC. The focuses of the present article are the experience and perspectives of 11 suicidal female adolescents, based on the richness of data obtained from interviews with them. Interviews were analyzed using Grounded Theory [87], and the analysis was guided by a thematic analysis [88]. The study protocol received ethical approval from CMO Arnhem-Nijmegen (Registration number 2019–5571).

### Participants

Adolescents aged 12–18 were recruited from three SRYC-institutes. Exclusion criteria included adolescents: i) with no adequate mastery of the Dutch language, ii) who had been suicidal shortly prior to the interview and therefore at high risk of severe distress, iii) who were unable to complete an interview of 40–60 minutes and iv) placed in the SRYC institution less than four weeks prior to the interview. We included 11 suicidal female adolescents, there were no dropped outs or refusals. All participants had previously been engaged in suicidal behavior. Moreover, nine participants had a record of self-harm of which cutting was the most used method. Except for one, all participants had a long, comprehensive history in youth care before placement in SRYC. Adolescents had multiple psychiatric diagnoses (ranging from autism, ADHD, depression, psychoses, anxiety, eating disorders, personality problems, substance use, adjustment problems and behavioral problems) which they received prior to placement in SRYC. In addition, all adolescents experienced a completed suicide by a group member while residing in SRYC. At the time of the interviews participants resided a minimum of 6 months to more than 18 months in the SRYC facility. One participant was 14 years of age, four were 15–16 years, and six participants were 17–18 years of age. For clarity’s sake, in this paper we refer to our participants as adolescents.

### Procedure

Adolescents aged 16 and over signed their own form of consent. For adolescents aged under 16, written consent from both (authoritative) parents/legal representatives or guardian was needed. Individual interviews were conducted using a semi-structured interview design. Open-ended questions and probing questions were asked to gain more detail on a particular issue. Interviews were conducted by a trained qualitative researcher (SK) and an experienced expert and lasted between 40 min and 1.5 hour. The trained experienced experts all had a social science degree (Bachelor or Master). All interviews were conducted face to face at the SRYC institute, except for one which was held at participant’s home. One participant was anxious to conduct the interview alone and requested for her mentor, with whom she had a bond of trust, to be present at the interview. A follow-up phone call was made the day after the interview. The participant confirmed that she had not been hesitant to speak openly because of her mentor’s presence. Moreover, her mentor’s presence helped her reduce her anxiety. As the presence of the mentor was no confounding factor, we did not exclude the interview results.

Adolescents were asked in advance if they had a gender preference for the experience expert. Prior to the interviews, the researcher discussed the study and the reasons for the interview. Also, prior to and during the interview it was emphasized that adolescents could stop at any time if they felt uncomfortable. Field notes were made from the researcher observations during and after the interviews. The interviews were audio-recorded with the adolescents’ consent and transcribed verbatim. All transcripts were anonymized. Adolescents were offered a transcript of the interview by email or post, and the interviewer followed up by phone to note any changes or comments. In the member-check, all adolescents agreed on the accuracy of the transcription of their interview and did not censor the content.

Given the sensitive nature of the subject, ample time was taken to get acquainted and establish a safe atmosphere (“rapport”) before starting the interview [89, 90]. In order to provide a safe and comfortable interview environment the experienced experts discussed their own life story and experiences prior to the start of the interview. The interviewer contacted each of the adolescents within three days after the interview to inform how they had perceived the interview and how they felt afterwards. In one case, the participant was transferred home after the interview and, for privacy reasons a group worker contacted the participant instead of the researcher. There was no need to repeat any of the interviews. The audio recordings were deleted to guarantee anonymity. Adolescents received a gift card after the interview. The Consolidated Criteria for Reporting Qualitative Research (COREQ) [91] was followed (see supplementary).

### Interview guide

Adolescents were asked how group workers reacted to suicidal behavior (including self-harm) and the impact of these reactions on the adolescent and the group climate. We also explored how adolescents reacted to suicidal behavior (including self-harm) of a group member. We used a semi-structured interview guide based on the following key themes:

- reason for being placed at SRYC;
- daily life at the residential facility;
- the reaction and attitude of group workers towards suicidal behavior;
- the opinion of adolescents on the reactions of group workers on suicidal behavior;
- impact reactions group workers on group climate / atmosphere;
- impact of suicidal behavior of group members on adolescents.

Probing questions were asked for more detail on a specific issue such as: “Can you explain that?”, “Could you tell me more about ….”, “How did you feel about that”, “What do you mean with…”. Each interview was conducted in the form of open communication between the interviewers and the participant. The interview guide was used mainly as a checklist.

### Sampling (Recruitment of participants)

A mixture of purposive and convenience sampling was used [90]. We recruited adolescents through a contact person at the SRYC institute, in close coordination with group workers and the responsible main practitioner. Interviews were held between October 2019 and December 2020. Data saturation was reached.

### Qualitative analysis

The study was performed in close cooperation with five experienced experts (four females, one male) who participated as co-researchers. Together with the interviewer (SK, female), the experienced experts conducted, transcribed, coded and helped analyzing the interviews. All interviews were conducted in pairs; this added value as when debriefing, the researchers were able to process the emotional intensity of the stories together. During the elaboration, coding and analysis, the researchers had intense discussions and conversations with each other, in which the co-researchers contributed their own experiences and in this way, were an additional source of knowledge. The perspective of the experienced experts gave further insights in the data [87, 92] and helped exploring and explaining adolescents’ views.

The transcripts of the interviews were analyzed using Atlas.ti version 8.4 (Scientific Software Development GmbH, Berlin, Germany). Themes were developed inductively, ensuring coding stayed close to the adolescents’ accounts and the issues important to them. In this way, coding was data driven instead of theory driven. After reading and re-reading to become familiar with the data of the first interviews, researchers started open coding of the data segments relevant for answering the research question. Each interview was coded independently by two researchers (the first author and one of the experienced experts), followed by comparing codes and discussing differences to derive inter-coder reliability [87]. The use of multiple coders increased the diversity of perspectives on our data. The next step of analysis involved grouping data with the same issues in one category. Categories were then combined to construct overarching themes [93]. Whenever necessary, new codes were added to the codebook or previously used codes were combined or deleted in case a better description of the data became evident. Earlier interviews were recoded when necessary. After 11 interviews, the researchers concluded that data saturation had been reached. The analysis centered around four main themes e.g., non-responsive reactions, responsive reactions, impact of the responses on adolescents, impact of the responses on group climate. Subthemes were identified within each theme. The final stage of analysis was reviewing the data to ensure themes and subthemes were sufficiently supported and to extract direct quotes illustrating the themes and subthemes. This analytical process occurred at several meetings with members of the research team. A research diary of the research meetings and discussions was kept.

## Results

**Fig 1:**
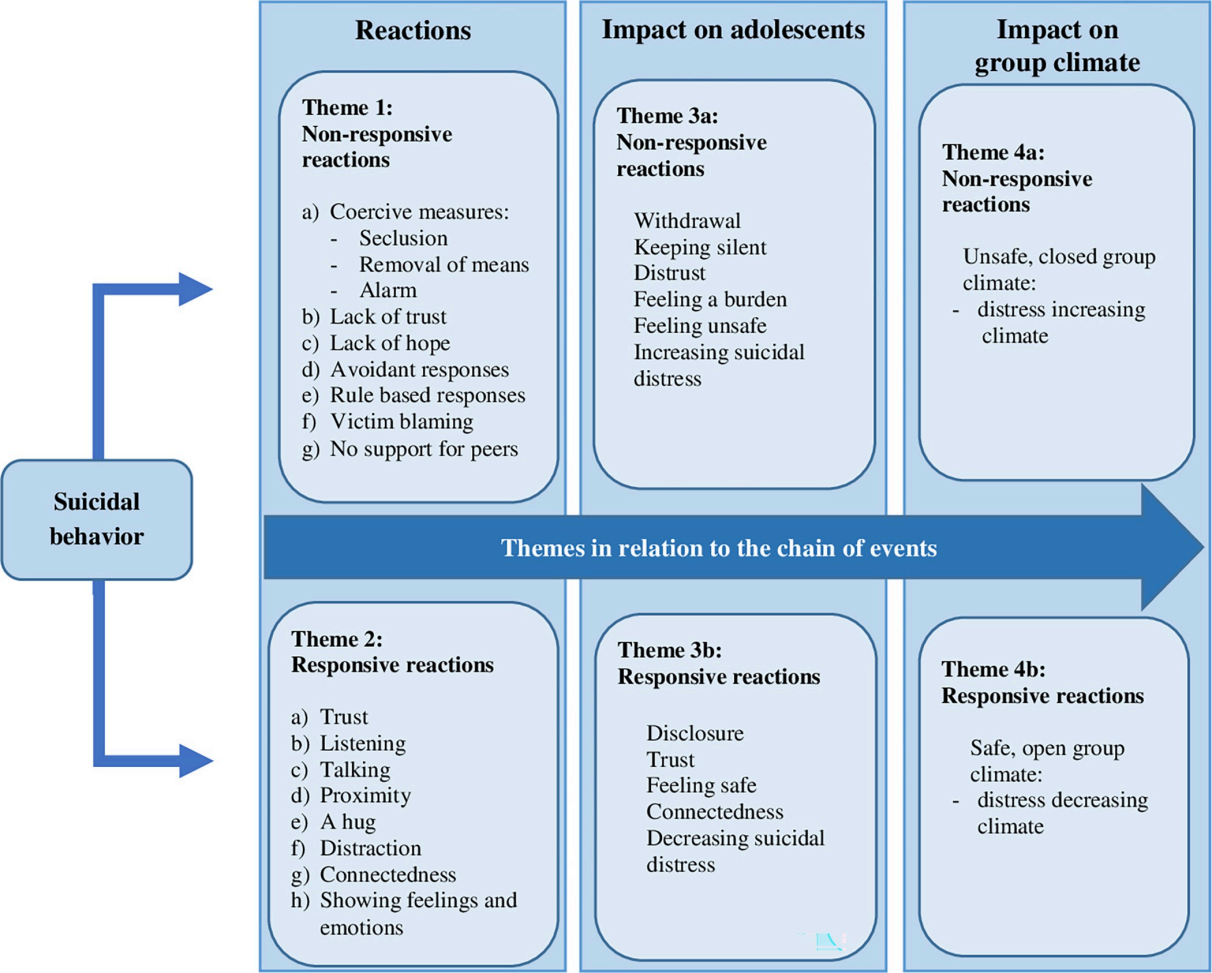
Chain of events: Themes and subthemes.

We conducted semi structured interviews with 11 suicidal female adolescents in SRYC to identify how they experienced group workers’ responses to suicidal behavior. We also explored the impact of these responses on adolescents and the group climate. Adolescents identified a range of positive as well as negative reactions to suicidal behavior, but basically, their stories revealed four major themes: 1) non-responsive reactions and 2) responsive reactions. The reactions of group workers affected the consequent interaction patterns on the individual as well as the group level, and resulted in the third and fourth theme: 3) impact on adolescents 4) impact on group climate. Themes were divided in subthemes who are discussed separately below. Quotes are translated from the Dutch. The themes in relation to the chain of events are illustrated in Fig 1.

### Theme 1. Non-responsive reactions

All adolescents gave multiple examples of non-responsive reactions of group workers to suicidal behavior. To provide more detail, the theme was subdivided into: a) coercive measures, b) lack of trust, c) lack of hope, d) avoidant responses, e) rule based responses, f) victim blaming, g) no support for peers. It is important to note that the subtheme “coercive measures” was given more space than other subthemes as all adolescents extensively discussed examples of coercive measures. In the subtheme ‘coercive measures’, the detrimental effects of involuntary seclusion stood out and were addressed by all adolescents. Adolescents stated that their indignation with the use of coercive measures in general, and involuntary seclusion in particular were important reasons to participate in this study.

#### a. Coercive measures

It is important to note that the subtheme “coercive measures” was given more space than other subthemes as all adolescents extensively discussed examples of coercive measures. Adolescents described how coercive, restrictive measures like involuntary seclusion, restraint and fixation, pushing alarm, removal of means, and not being permitted to go outside were used by group workers in order to control and stop suicidal behavior. In the subtheme ‘coercive measures’, the detrimental effects of involuntary seclusion stood out and were addressed by all adolescents. Notably, all were moved and visible emotional when discussing their experiences. Adolescents stated that their indignation with the use of coercive measures in general, and involuntary seclusion in particular were important reasons to participate in this study.

*Coercive measures*: *Seclusion*. The majority of adolescents stated that if talking about their suicidal thoughts led to involuntary seclusion, they chose to remain silent and keep their suicidal feelings a secret. Adolescents described how coercive, restrictive measures like involuntary seclusion, restraint and fixation, pushing alarm, removal of means, and not being permitted to go outside were used by group workers in order to control and stop suicidal behavior. Adolescents reported that the negative effects of coercive measures in general and involuntary seclusion in particular, ranged from anger, despair, and sadness to loss of hope.

Most adolescents spoke openly about their experiences with involuntary seclusion, which often had a negative and traumatic impact. Hence, two adolescents were unable to talk about the subject as it was too stressful for them. One participant was willing to discuss the subject but choose to describe her experiences in a more superficial, distant way in order to avoid severe distress. Others were moved and emotional when discussing their experiences with involuntary seclusion. Some adolescents spoke of feelings of shame and humiliation, others spoke of despair and feeling abandoned and punished. Notably, a common feeling described by all adolescents was anger and indignation about being restraint and secluded. For some being involuntary secluded felt as being punished. Some adolescents were surprised that group workers actually seemed to believe that involuntary seclusion was a helpful intervention for decreasing suicidal behavior. According to adolescents the opposite was the case and they advocated that involuntary seclusion perpetuated and aggravated their suicidal behavior. Adolescents stated that once in an isolation cell, the lack of distraction reinforced their suicidal thoughts, as described in the next quote.

“*In isolation*, *when you’re sitting between those four walls*, *you are so alone*, *and the suicidal thoughts only get worse and worse*. *It makes you want to do it even more*. *If talking has all these negative consequences*, *then you must be crazy if you told them you are suicidal*.*” (D37*, *girl age 17)*

None of the adolescents said to have benefited from involuntary seclusion. According to them, rather than secluding youth in crisis situations, it would be more helpful to interact with adolescents in order to decrease the increasing distress. Some adolescents described group workers as being uninvolved and distanced. They mentioned a lack of communication when being put in seclusion. Hence, being isolated exacerbated feelings of being a burden, which made some adolescents feel not being worthy of asking for help. Being isolated felt like being abandoned, even if a group worker would come and check every fifteen minutes.

*“Once put in isolation the feeling I got was, ’they don’t want to spend time on me’. Yeah, they check on you every 15 minutes, but they don’t ask you anything. Nobody talks to you. I felt that I was a burden to everyone. It was my own fault; I was the cause of this all. So, I was not worthy of asking for any help. Anyone would think that once you’re put in isolation. It only made things worse.” (D35, girl, age 16)*

In sum, the majority of adolescents did not approve of the use of isolation cells as a response to suicidal behavior. Some adolescents reflected on the impotence and fear of group workers and indicated that involuntary seclusion seemed to be used out of ignorance, as group workers did not know how to handle suicidal behavior otherwise.

“*There are situations in which someone is acute suicidal and takes every opportunity to kill herself*. *Hence I understand that they put someone in isolation*. *Don’t get me wrong*. *Isolation is never good*. *Never*. *But at times I understand that they do use it*, *because obviously they don’t know what to do otherwise to keep someone safe*.*” (D35*, *girl*, *age 16)*

Although not condoning the use of involuntary seclusion, two adolescents gave suggestions for improvement to reduce the negative effects of involuntary seclusion. They urged group workers to provide adolescents with mental help and support in order to minimize collateral damage. So instead of using violence to restrain an adolescent in crisis, group workers should be more kind and human and explain why they were using involuntary seclusion.

“*It is better not to use forced isolation*, *just don’t*. *If you [raises voice] really really must use it then you ought to provide help for the mental part when you isolate someone*. *It could just be a lot more loving*, *instead of pressing alarm and fighting with you*. *That makes it even harder and worse*. *Say what you are going to do and why you do it*. *Be human*, *show love*, *give a hug and be supportive*, *be there for the person*. *That really can be improved*.*” (D37*, *girl*, *age 17)*

Although the majority of adolescents spoke about the many negative effects of involuntary seclusion on themselves as well as on their relationship with group workers, one participant described an exception. Participant D35 recalled a group worker who was warm and gentle while putting her in an isolation cell. This warm reaction surprised her.

“*The first time I ended up in a solitary cell I was totally upset and didn’t know what hit me*. *The group worker who put me there*, *did it alone*. *Normally there is a bunch of group workers who put you there*, *but she did it alone*. *She literally tucked me in with my own blanket in order to calm me down a little bit*. *She had to put me there*, *because of the protocol*. *But I will never forget the caring way in which she put me there*.*” (D35*, *girl*, *age 16)*

*Coercive measure*: *Removal of means*. All adolescents had experience with potentially harmful objects being taken away in order to prevent them from harming themselves. They explained that removal of means may seem a logical step to achieve safety. However, the majority underlined that taking away sharp things increased suicidal distress and often triggered them to self-harm even more. Stripping a room was perceived as a very intrusive measure which often resulted in a power struggle between adolescents and group workers. By contrast, this could make the situation more dangerous and life-threatening. This was especially the case when adolescents did not have an alternative to cope with their distress otherwise. Hence, out of fear for removal of means, adolescents often lied about their urge to self-harm and hide sharp objects. Dangerous situations could then arise as described in the next quote.

“*A girl had swallowed a razor blade last week*. *She had to go to the hospital*. *I spoke to her when she came back*. *It was an accident*. *She did not wanted to die or so*. *She had had a razor in her mouth for weeks*. *Because she was afraid it would be found*.*” (D20*, *girl*, *age 17)*

According to the majority of adolescents removal of means was not helpful when being distressed and feeling the urge to self-harm. There were again suggestions for improvement. Group workers should offer distraction and proximity instead by calmly talking to adolescents to help adolescents in using other coping mechanisms than self-harm to regulate distress. Participant D35 had wished for a group worker who would had praised her for being able to postpone self-harming, or who was familiar with distraction techniques and alternative coping mechanisms. Adolescents emphasize that ’good behavior’ must also be given attention. In the next quote she described why such an approach would had been more helpful to her than being involuntary secluded.

“*It doesn’t help to take away sharp things and stuff*. *There is always something I can find to harm myself*. *If you lock me up it really gets dangerous as I’ll just do crazier things to escape these negative feelings*. *Taking pills and stuff*. *I don’t want to die*. *But I can’t cope with the distress*. *Instead of removing sharp things or seclude me*, *you’d better stay with me*, *talk to me or do something to distract me from the distress in my head*. *And also*, *if I have been able to postpone the cutting; instead of saying “you did it again*, *that’s wrong*!*” It would be so much better if they praise me that I’ve been able to delay it for a while and therewith give me hope that I can change my behavior*. *But instead they put me in isolation*.*” (D35*, *girl age 16)*

*Coercive measure*: *Alarm*. Adolescents described how the atmosphere got tensed when group workers pressed alarm for assistance if they could not handle incidents alone. Group workers from other groups would rush in to offer assistance. These incidents in itself caused fear and panic among group members. Adolescents underlined how stressful it was to witness a peer being restrained and put in isolation while having a predisposition for suicidal behavior yourself. Witnessing these incidents was overwhelming and triggering as it made adolescents feel unsafe and anxious. Hence, for some adolescents alarm being pushed it had a huge negative impact on their own distress. Participant D14 was visibly moved and emotional when discussing the huge impact of alarm being pressed.

“*It’s very scary when you see a lot of people come barging in*, *and someone is thrown ’bats*!*’ on the floor*. *Ohh*, *I really can’t see that*, *it makes me panic*! *For me it’s really traumatic*, *when I see that*, *I totally panic*. *I get re-experiences of what is was like for me when that happened with me*. *And the screaming*! *I just cannot bear to hear someone scream while being dragged all the way to isolation*. *"Let go of me*!*"*, *I really can’t hear that*, *I know how that feels*. *I panic completely and get triggered myself*.*" (D14*, *girl*, *age 17)*

#### b. Lack of trust

The majority of adolescents expressed a lack of trust towards group workers. At the same time they highlighted that in order to disclose, they needed to trust a group worker. Adolescents mentioned several reasons that hindered them to gain faith in a group worker. First and most importantly, adolescents distrusted group workers out of fear for coercive measures when discussing their suicidal feelings. As a result, they remained silent. Secondly, spending time together was an important factor to get to know each other. However, the necessary time to build on a bond of trust was often lacking which was perceived as an obstacle to gain trust. Finally, privacy issues were mentioned as adolescents felt embarrassed when group workers told peers about suicide attempts or incidents without their permission to do so, which did not added to the trustworthiness of group workers. All in all, being confronted with coercive measures in response to suicidal behavior stood out as the most important cause of mistrust among adolescents.

“*I would have preferred that they had talked to me instead of secluding me*. *Because I really didn’t needed to be isolated as the cutting was already done and the tension was gone*. *So there was no need to seclude me*. *But they did*, *as they said I still was a danger to myself*. *’Being safe for the night’*, *[in a cynical voice] that’s what they called it*. *But they didn’t talk to me nor asked me anything*. *While I knew I wasn’t a danger to myself anymore*. *But they would not talk to me nor listen*. *They just locked me up*. *So how can I ever trust these people*? *I can’t*, *I simply can’t trust them*.*” (D34*, *girl*, *age 16)*

#### c. Lack of hope

A number of adolescents stated the need for more future perspective and meaningful daytime activities in order to recover from depressive and suicidal feelings. Nonetheless, according to the majority of adolescents these were offered too little in SRYC. School, and thus having a future perspective, played an important role but often school did not match the academic level of the adolescents. Hence, more attention should be paid to the talents of adolescents in SRYC and more should be done to provide hope for a better future. Moreover, adolescents described a lack of understanding about the impact of living in a group with other suicidal peers. The majority of adolescents indicated that they became unbalanced themselves due to suicidal incidents which often had a "contagious" character. In addition, adolescents underlined that their placement in a depressing living environment with peers who all had serious problems, did not contribute to overcoming their own problem behavior. As a result some lost hope for a better future.

“*How can you get better in a group where everyone is depressed and suicidal*? *You’d better be having positive people around you so you are inspired to make something of your life*. *So you get hope that you can solve your problems*. *It is wrong to put all these people with serious problems together*. *I miss my normal life and the ordinary things*. *In here we only talk about problems*. *How can you build your life in here and move on*??*” (D37*, *girl*, *age 17)*

#### d. Avoidant responses

Adolescents described how some group workers reacted avoidant in response to suicidal behavior. The majority of participants stated that suicidality in general should be discussed more often. However, according to some adolescents, most group workers were not well educated for knowledge on how to discuss these subjects seemed to be limited. Some adolescents argued that it would be better if group workers pro-actively talked about suicidality thereby breaking the taboo surrounding the subject.

However, most group workers waited for adolescents to take the initiative to address the subject. When adolescents did disclose on their suicidal feelings, they needed group workers to demonstrate understanding and empathy, instead of responding avoidant, blaming or angry as often was the case. Participant D37 described how, instead of receiving mental support after a suicide attempt, she was faced with silence. She missed being able to talk about what happened which added to her feelings of loneliness.

“*I made a suicide attempt at the group*. *The attempt failed*, *but it was simply not discussed*, *nobody talked about it*. *I was placed in an isolation room for solitary confinement*, *which was horrible*. *And the next day everybody would act as if nothing had happened and we would be having breakfast all together*.*”*

*Interviewer*: “*And how did they talk with you about your suicide attempt*?*”*

“*After an attempt they should [raises voice] talk to you*, *but nobody talked about it*. *It made me feel very alone*.*” (D37*, *girl*, *age 17)*

#### e. Rule based responses

According to the majority of adolescents, life at the living group was characterized by a huge amount of rules. All adolescents agreed that rules were needed to structure daily life. However, some adolescents described a lack of fairness, flexibility or predictability of the existing rules. The vast majority of the adolescents perceived a lack of possibilities to influence rules or to decide together when in crisis, which resulted in feeling powerless and not being listened to. Some adolescents underlined the inequality between group workers and youth in SRYC in terms of power. Hence, adolescents welcomed a greater involvement in decision making which would have increased their sense of being listened to. However, often the application of the existing rules seemed to vary and it depended on the person employed how an incident was assessed and what measures were taken. As a consequence the variation in application of rules could be large, varying from very loose to an overly rigid application of rules which could provide an indirect risk for individuals to escalate.

*Interviewer*: “*How is your security plan used*?

“*Depends on who is employed*. *One trusts me and knows me better*, *so suppose I cut myself or something*. *My signaling plan states ’don’t go along with emotion*, *just give a band-aid’*. *And others also know me*, *and trust that I hand in whatever sharp things I have*. *Some don’t trust me at all and place me in comfort [comfort = isolation room*, *SK]*. *One puts me in comfort with my own clothes and the other puts me there in a rip dress*. *So it really depends on who is employed*. *And that makes me so angry sometimes*, *they can do whatever they like*. *Sometimes I lose it when I get angry and things really escalate*.*” (D14*, *girl*, *age 17)*

#### f. Victim blaming

Adolescents described how some group workers blamed adolescents for self-harming. Others became angry and openly judged adolescents’ suicidal behavior to be manipulative or attention seeking behavior. This resulted in adolescents feeling guilty and ashamed. Others felt not acknowledged in their struggle and withdrew as a result. While some group workers were profoundly disapproving of adolescents who self-harmed others would ignore problematic behavior or downplayed the severity of the suicidal condition of adolescents, for instance by refusing to take care of wounds. Adolescents judged these reactions to be careless and harsh. In the event of problematic behavior adolescents felt that group workers should act supportive and caring instead of reacting harsh or avoiding.

“*I remember I had been cutting myself and … [silence]…*. *the wounds were really bad and needed stitches*. *But they said*: *’Well*, *you may find out for yourself how to take care of this*.*” They gave me bandages for the wounds and just left*. *I had to bandage the wounds myself*, *but that’s a little hard to do with only one hand*.*” (D6*, *girl*, *age 16)*

“*They reacted harsh and negative after I self-harmed*. *When I pressed the intercom they came and said [sigh overly audible] "Ok*, *that again" and then they went to the first aid cabinet to take some bandages and throw them in my room and say "good luck*!*" and then I would bandage myself*.*” (D41*, *girl*, *age 18)*

#### g. No support for peers

A large number of adolescents stated that living groups in SRYC were too large and the number of group workers too small. Hence, it was impossible to pay sufficient individual attention to all group members when an incident occurred. Adolescents described their mounting distress and anxiety when witnessing an incident at the group. However, group workers were busy taking care of the adolescent in crisis, and often there was no additional staff present to minimize the disruption to the other group members. As a result, incidents in the living group often triggered similar self-harming or suicidal behavior amongst other group members.

“*I find it very difficult to ask for help as it is*. *Sometimes I manage to do so*, *but then no help can be given as group workers are too busy taking care of the adolescent in crisis*. *The alarm*, *the panic*, *fixations and all that really upsets me*, *and then I get really confused with myself*. *During an incident I don’t know what to do anymore*, *and things get out of hand completely*. *For it triggers me to witness someone being fixated*. *I get so tensed and I just lose myself*. *I often end up cutting myself as a result*.*” (D14*, *girl*, *age 17)*

### Theme 2: Responsive reactions

In this theme a considerably smaller amount of adolescents described recalling responsive reactions to suicidal behavior by group workers. In order to understand the core content of this theme we divided it into the following sub-themes: a) trust, b) listening, c) talking, d) proximity, e) a hug, f) distraction g) connectedness, h) showing feelings and emotions.

#### a. Trust

All adolescents stressed that trusting a group worker was a precondition to disclose on suicidal feelings. Spending time together was helpful in order to get to know and eventually to be able to trust a group worker. A friendly approach, a calm, soft tone and a sensitive interaction style was crucial for building a bond of trust. Sincere listening, being honest, and paying attention were also important features for adolescents to gain trust. Adolescents’ view of group workers who offered these kind of responsive care was positive and they valued having good, supportive relationships with these group workers. An adolescent described how she trusted and confided in a group worker which resulted in her handing over sharp things voluntary.

“*My mentor is caring and gentle*. *She doesn’t get angry with me when I have cut myself*. *She helps me bandaging my wounds and stuff*, *and she shows compassion*. *When I need stitches*, *she always stays with me at the hospital*. *She doesn’t judges me and that makes me feel safe with her*. *We have such a good relationship by now that I confide in her when I have sharp things in my room*. *She makes me hand them over to her*. *I don’t know how she does that*, *but I do [laughs]*. *I really trust her*.*” (D6*, *girl*, *age 16)*

Participant 32 described how she asked for help after having cut herself. She was put in isolation. However, this intervention was performed based on collaboration and mutual trust. The existence of a strong, positive relationship with her group workers had an impact on the whole situation, of which voluntary seclusion as well as the adolescent’s need for safety and security was a part. Hence, by discussing the situation together it was determined how the situation had to be handled. This approach worked out well.

“*I don’t know*, *I just trust them here*. *I recently have cut myself*. *I pressed the intercom and asked for help*. *And they helped me*, *they just listened to me and put me in control*. *I didn’t wanted to sleep in my own room*. *And we talked about it*, *why I was feeling so bad and all*. *I chose to sleep in the safe room myself*. *It felt better also because I made that choice myself*. *The next day I just went back to my own room*. *They did not remove sharp things or stuff from my room*. *They trusted me and we just picked up the pieces and went on*.*” (D32*, *girl*, *age 14)*

#### b. Listening

Adolescents underlined the importance of a group worker who listened calmly and acknowledged their suicidal feelings and distress without becoming judgmental, angry or taking over control. The ability of group workers to listen with patience and emphatic understanding without judging or attempting to provide premature reassurances helped adolescents sort out their feelings. When being suicidal or having the urge to self-harm some adolescents argued, you often do not know exactly what is going on in your mind that causes all the suicidal distress. As a result of the mounting distress some adolescents could literally find no words to describe their suicidal feelings. It was helpful when a group worker acknowledged and accepted this inability and helped adolescents to calm down first before exploring what caused the distress.

“*How are you*?*” Is a very difficult question*. *It is way too broad*. *Then I answer something like ’I am ok’*. *Because often I just don’t know why I am so tensed and upset*. *If I knew I would say it*. *When a group worker just listens and respects that I don’t know what is going on*, *I calm down*. *That helps*. *Then my tension drops and we can unravel what actually happened that made me feel this way*. *There is always a reason why I want to hurt myself*, *but I am unable to see that it if I am so stressed out*.*" (D35*, *girl*, *age 16)*

#### c. Talking

Adolescents emphasized that talking about suicidality with someone trusted relieved tension and reduced suicidal distress. While considering that talking, offering support and unconditional help were beneficial for participants, the interview data suggested that in the majority of facilities were the adolescents resided these forms of support were rare. However, there were exceptions. The adolescent in the next quote resided at an SRYC facility where residents themselves could unlock their doors at night and ask for help. Group leaders were available 24 hour per day which enabled the adolescent to ask for support during the night. At this facility no coercive measures were used the moment an adolescent expressed her suicidal feelings or in response to suicidal behavior. Group workers offered relational safety instead. According to adolescents the atmosphere at the group was open and friendly. Participant D35 described how this ability to talk freely about her suicidal feelings was beneficial to her.

“*At night there is always a group worker who sleeps at the group*. *That gives me a safe feeling and makes it possible for me to reach and ask for help*. *I often sat in the middle of the night with a group worker to talk*. *When things really went wrong one time [participant attempted suicide*, *SK] I was so afraid that I would be transferred or that I would end up in isolation and stuff*. *They said “no*, *we are not going to do that”*. *The ability to freely talk about my feelings really helped me when I felt suicidal*. *(D35*, *girl age 16)*

#### d. Proximity

Adolescents described the positive, preventive effect of a group worker staying near and offering proximity when being in a suicidal crisis. Adolescents highly appreciated group workers who stayed unconditionally during a suicidal crisis, even if the adolescent was not able to speak or make contact. Group workers who remained calm and supportive, were highly valued. Knowing not to be not left alone in times of suicidal distress, but instead being acknowledged and supported while in pain and distress gave adolescents a sense of safety and reassurance. According to adolescents, this kind of proximity was helpful during a crisis and decreased suicidal feelings. Not being left alone, but being cared for and looked after in bad times was helpful to get through a suicidal crisis as described by participant D37.

“*A very positive experience for me was that I had a group worker who just came to sit in my room with me and he literally sat through my crisis with me*. *He said "I’ll stay with you and we’ll do this together"*. *I often dissociate and then I don’t know what I’m doing anymore*, *so it’s very helpful when you know that someone stays with you*. *That you know you are safe*. *That really helped me to overcome my crisis without harming myself*.*” (D37*, *girl*, *age 17)*

#### e. A hug

All adolescents appreciated a hug when having a hard time. However, adolescents only wanted a hug from group workers they trusted and felt at ease with. Cuddles were important for hugs had a huge calming effect on adolescents’ suicidal thoughts and mental distress. A hug made adolescents feel connected and being cared for and dispelled feelings of intense loneliness. The beneficial effect of a hug was mentioned by all participants. In many institutions however, it was forbidden to give hugs or make physical contact. Although forbidden, some group workers gave hugs anyway. By doing so they sometimes prevented things getting seriously out of hand, as described in the next quote.

“*At home I would get a hug from my parents*. *In here*, *I would not get a hug from a social worker*, *because that is not allowed in here*, *because of the protocol*. *I have two group workers with whom I had a good connection*. *They were a bit [whispering] rebellious and just gave me a hug anyway*. *A hug is one of those little things that really mean a lot*. *I know for sure that getting a hug prevented me several times from harming myself and it prevented things getting completely out of hand*.*" (D37*, *girl*, *age 17)*

#### f. Distraction

All adolescents emphasized that offering distraction was beneficial in lowering suicidal distress. Adolescents preferred group workers who helped seeking distraction over group workers who simply told them to stop their suicidal behavior. Talking calmly, making contact and deciding together what to do, was perceived as a successful distraction technique in times of crisis. Most adolescents had their own tailor made safety plan which described what was helpful in times of distress. Adolescents preferred a group worker who would not just follow the safety plan, but who consulted the adolescent first. The more adolescents were involved in deciding what kind of distraction would be helpful to change their situations, the greater their sense of personal control. Choosing together, instead of being told what to do was helpful in decreasing suicidal distress.

*Interviewer*: “*What helps you when you are in distress*?*”*

“*That they think along with me about what might be helpful and that they let me choose*. *For it helps me that it is possible to offer the distraction that I choose at that moment*. *For distraction really helps me and being able to choose is beneficial too*. *For me*, *for example*, *playing a game of football helps me when I am suicidal*.*” (D31*, *girl*, *age 16)*

#### g. Connectedness

Adolescents emphasized the importance of feeling connected to a group worker, which was strongly intertwined with trusting a group worker. Connectedness and trust enabled adolescents to ask for help when feeling depressed and suicidal. According to respondents, a group worker who was genuine and showed something personal of himself made the relationship more equal and was easier to connect to. Adolescents preferred group workers who would “loosen up a bit” in their interactions and who were present and available in the living group instead of staying in the office. This included group workers who expressed confidence in adolescents and adjusted rules accordingly. “Just being a normal person, being yourself” made a group worker more trustworthy and therewith easier to turn to when feeling bad.

“*One group worker is just relaxed and fun to be with*. *Others just stick to the rules*. *“No eggs during the week*!*”*. *And that’s it*. *But he*, *he is chill*. *“Would you bake an egg for lunch on Wednesday*? *Fine with me*, *let’s do so*.*” He hangs out with us on the couch*, *gaming on his phone*. *But if you want to talk to him*, *he is just there for you and you really feel connected*. *He just listens*. *We all turn to him when feeling bad*. *When he works*, *the atmosphere at the group is really nice*. *Relaxed*. *(D31*, *girl*, *age 16)*

#### h. Showing feelings and emotions

Adolescents highly appreciated group workers showing feelings and emotions. Group workers not hiding their feelings and emotions behind their role as professional, were considered authentic and trustworthy. Sharing and being open about yourself, could concern small everyday things but could also entail emotions to an adolescent’s suicidal struggle or incidents. In the next quote participant D3 described how she saw the emotions of a group worker after he had experienced a serious incident. This observation made her realize that he was human too which made it easier for her to ask his help when she felt suicidal herself.

“*Well*, *he [the group worker] returned after he provided assistance at another group because of a suicide attempt*. *I saw that he was upset and had tears in his eyes*. *I didn’t like to see that*. *But I realized that he was moved by the incident and that he is only human too*. *Well I just really see him now as a… well [hesitates] … it sounds very strange … as a real person with feelings*. *Because with most group workers you just don’t really see that they are human and have emotions too*. *It just made it easier for me to connect to him and go to him for help when I was suicidal myself*.*” (D3*, *girl*, *age 15)*

### Theme 3a: Impact non responsive reactions on adolescents

Adolescents were very clear on the negative impact of non-responsive care in general (getting angry, victim blaming, raising voice, responding avoidant, lack of communication etc.) and coercive responses (involuntary seclusion, restraint, camera surveillance, etc.) in particular. Out of fear for involuntary seclusion, adolescents did not dare to talk about their suicidal feelings and remained silent. Judgmental or disapproving reactions of group workers resulted in adolescents feeling a burden which reinforced feelings of helplessness and increased suicidal distress. The impact of coercive responses ranged from despair, feeling unsafe, sadness to loss of hope which could result in feeling unconnected, spiraling down and suffering alone. These feelings ultimately resulted in withdrawal and an increase of suicidal feelings.

”*The point is*: *If they know I have a death wish*, *then there are so many consequences*: *Then I have to go to the safe room or the ISO [isolation room*, *SK]*. *And then I am being watched all the time*. *And that makes my death wishes only worse*, *and I end up just wanting to do it*, *right*? *They mean it as help*, *but for me it feels like punishment*. *So I usually keep my suicidal thoughts to myself*.*” (D31*, *girl*, *age 16)*

### Theme 3b: Impact responsive reactions on adolescents

Group workers who reacted responsively to suicidal behavior had a positive, decreasing impact on suicidal distress of adolescents. Being able to trust a group worker, being taken serious and having a bond of trust with a group worker encouraged adolescents to disclose on their suicidal thoughts which reduced their suicidal distress. All adolescents valued group workers who handled incidents by setting limits without using coercive measures or punishments but who offered emotional support instead. Emotional support is featured by listening and talking calmly, being available and staying near. Being cared for, listened to and shared decision making gave adolescents a sense of trust and control. Participant D32 described how having positive relationships with her group workers made her feel safe. This was her starting point for asking help and talking about her suicidal feelings. Over time, this helped her in her recovery.

“*I was suicidal when I entered here one and a half year ago*. *At that time*, *it was it really difficult for me to ask for help when having these suicidal feelings*. *And now*, *a year and a half later*, *a lot has changed*. *I feel safe here*, *and I have faith in the group workers around me*. *I have a good relation with my mentor*. *They listen to me and mean well in here*, *and I talk much more now than I did at first and I dare ask for help when I don’t feel well*. *And I go to school*, *I focus on my future*.*” (D32*, *girl*, *age 14)*

### Theme 4: Impact on group climate

The impact of suicidal behavior and incidents on the living environment is significant. However, the magnitude of this impact depended on the way group workers responded to the suicidal behavior. Analyzing adolescents’ reports resulted in two subthemes: (a) unsafe, closed group climate (b) safe, open group climate.

#### a. Impact non-responsive reactions on group climate

Adolescents described how non-responsive reactions in general and the use of coercion in particular provoked anxiety, distress, anger and distrust. This resulted in a chain of events in which adolescents perceived the negative atmosphere in the group climate as a direct consequence of the way group workers reacted to incidents. Adolescents described how tension rose when coercive measures were taken and as a result of this mounting tension other group members started displaying problem behavior as well. This again had a negative effect on the atmosphere in living groups and so on. In sum: Suicidal behavior and incidents in living groups increased tension and stress amongst residents. This tension and stress tended to increase when coercive measures were used in response to suicidal behavior and incidents. Ultimately non-responsive reactions to suicidal behavior and incidents led to an unsafe, closed group climate.

“*I often experienced that when staff had to separate and isolate a group member there would not be enough staff to take care of the rest of the kids*. *While in the meantime the group was going crazy because of the tension the incident caused in the group*. *Under while I was hurting myself in my room because I could not handle all the stress caused by it*.*" (D37*, *girl*, *age 17)*

#### b. Impact responsive reactions on group climate

The magnitude of the impact of suicidal behavior on the living environment and the disruption to group members could be minimized if group workers reacted in a responsive way to suicidal behavior. When group workers responded calm and responsive to suicidal behavior, the stress among adolescents decreased. This contributed to a safe and positive group climate. By offering relational safety instead of resorting to coercive measures, group workers limited the negative impact of suicidal behavior and incidents and contributed to feelings of safety. In sum: responsive reactions to suicidal behavior added to a positive group climate as stated by participant D19.

*Youth*: “*We also have other suicidal girls at the group right now*. *But even if there have been incidents*, *we still have fun together and there is a good atmosphere*. *Not a day goes by without laughing*.*”*

*Interviewer*: “*What might be the reason for that?*”

*Youth*: “*I think it is the fact that people here actually understand what you’re going through*. *That in here they understand if you’re having a bad day and they just… listen to you… accept it*. *I find it very hard to talk about myself and my suicidal thoughts*. *But in here I have talked a lot about my suicidal feelings*. *That helps me*. *I need people I can talk to*. *That it is important for me and in here adolescents as well as group workers really get along well together*. *Staff also occasionally go out together*. *Basically*, *we are just like a family together*. *That’s how it feels*.*" (D19*, *girl*, *age 17)*

## Discussion

This study explored how adolescents in SRYC perceive group workers’ responses to suicidal behavior. Group workers play an important role in the well-being and functioning of suicidal adolescents as they interact with adolescents on a daily basis. The aim of this study was threefold: (a) to explore how adolescents in SRYC view group workers responses towards suicidal behavior and (b) how these responses affect adolescents as well as (c) the group climate. As far as we are aware, we are the first to conduct a qualitative study that provides a rich systematic exploration of this topic from participant first person accounts. The findings can be used to develop care-policy to improve care for suicidal adolescents in SYRC.

We used the self-determination theory (SDT) to interpret the main findings. SDT distinguishes three different psychological needs which are strongly intertwined with the needs expressed by the adolescents in this research; autonomy, competence and connectedness [60]. In the interviews, adolescents underlined that they experienced a lack of connectedness. Moreover, they stated that taking over autonomy and control (lack of autonomy) by using coercive measures (e.g. fixation and isolation) harms them and increases suicidal feelings. Furthermore, the majority of adolescents criticize the lack of future perspective and hope (lack of competence), which is crucial to overcome their suicidal behavior. In sum, based on the findings in this study, and in line with previous research [57, 63], a lack of the three basic psychological needs may lead to an increased suicide risk in adolescents. Therefore, we stress the importance of awareness of the basic psychological needs postulated by SDT in order to affect psychological functioning and well-being of adolescents in SRYC.

The findings of this study underline the negative, detrimental impact of the use of involuntary seclusion. Besides feelings of shame, humiliation, and despair a common feeling described by adolescents was anger. Involuntary seclusion led to withdrawal, distrust, and non-disclosure of the interviewed adolescents, which reinforced feelings of solitude and isolation. In all cases, involuntary seclusion increased the suicidal feelings of adolescents. Out of fear for involuntary seclusion, adolescents refused to talk about their suicidal feelings. The findings of the present study lend support to previous findings in this area [44–46] and underline that seclusion endangers the therapeutic relationship. In addition, the use of involuntary seclusion can be a risk factor for exacerbating traumatic symptoms, which is consistent with previous research [94]. Our findings also reflect those of De Valk et al. [38] who found that using coercive, controlling measures results in a loss of connection with the young person, leaving two important psychological needs of the SDT, i.e. the need for connectedness and autonomy, unmet [59]. Ultimately, this can increase suicidal thoughts and behavior [35] as no contact with a suicidal adolescent is dangerous and increases the risk of suicidal incidents [47]. The awareness of these detrimental effects has led to efforts to reduce and prevent the use of seclusion in SRYC facilities, both in the Netherlands [23, 24] and worldwide [95–97]. However, our findings show that despite these efforts, involuntary seclusion is still commonly used in SRYC organizations in the Netherlands [42, 98].

The findings of this study also indicate the need for relatedness, proximity and trust in order for adolescents to disclose. Talking about suicidal feelings enables adolescents to regain some form of control, therewith meeting the need for autonomy and competence. Disclosure decreases the suicidal feelings of adolescents. This finding is supported by research of Dazzi and colleagues [48] who also found that acknowledging and talking about suicidal feelings reduces, rather than increases suicidal ideation. All adolescents appreciated responsive reactions such as a hug from a trusted group worker when having a hard time. A hug made them feel connected [62] and cared for, which had a calming effect on suicidal feelings and thoughts. However, in many SRYC institutions, it is forbidden to give hugs or make physical contact. This is partly due to recent studies reporting on sexual abuse and violence in youth care institutions [99, 100]. As a consequence, professionals are reluctant to discuss ’intimacy’, ’sexuality’ and ’professional distance and proximity’. Moreover, these themes are only marginally addressed in the basic curriculum training of professionals [100]. We therefore recommend that they are included in the training curriculum of professionals. Furthermore, we note a limitation of our research: it is not clear to what extent the need for a hug can be generalized to boys or to adolescents who are the victim of sexual abuse, as being touched can also be a trigger for victims of sexual abuse [101]. Further research on this topic is recommended.

An important aim of treatment in SRYC is to guarantee safety and to prepare residents for returning to and participating in society [79]. We therefore expected residents to benefit from therapy and education. However, the majority of adolescents experience insufficient opportunities for personal growth, and insufficient emotional and developmental support. School played an important role for adolescents but often did not match their academic level or was inadequately provided for. Furthermore, the majority of youngsters experienced too few opportunities within the closed residential youth care setting to develop and take on challenges for meaning full activities. They stated that more attention should be paid to the talents of adolescents which is done too little, leaving the need for competence unmet [62]. Drawing from self-determination theory (SDT), in the context of treating suicidal adolescents, attending school and focusing on future perspectives can reduce psychological suffering and may have a positive impact on intrinsic motivation for treatment [59, 102] which is important to achieve positive care outcomes [27, 103]. Adolescents would welcome being more involved in the decisions about their personal safety and their future. In doing so, it would increase their responsibility and satisfy their need for autonomy and therewith contribute to increase adolescents’ treatment motivation [104].

Findings show that the direct social environment of adolescents in SRYC, namely large living groups, may provide an indirect risk for suicide. Moreover, given the problem behavior of adolescents, which may involve a mix of disorders, living in a group with other suicidal group members was very complicated and experiencing suicidal incidents of group members was a barrier for overcoming their own suicidal behavior. The majority of adolescents were vulnerable when confronted with negative incidents and stressors. Adolescents stated that—although the impact of incidents on adolescents and the living environment was significant—the magnitude of that impact depended on the subsequent reactions of group workers. Group workers responding responsive reduced suicidal distress and therewith contributed to a positive group climate. The experiences of adolescents regarding sensitive, responsive reactions of group workers also illustrate how targeting the three basic needs postulated by SDT [59], helped adolescents to regulate severe distress and suicidal behavior. These findings suggests that adopting a responsive, strength-based approach, which respects the adolescents’ need for relatedness (in which contact remains when in a suicidal crisis), competence and autonomy, may help reducing suicide risk and recurrences of suicidal crises. However, non-responsive reactions increased tension among adolescents. This increased tension exacerbated the already complex working and living conditions and contributed to a closed group climate. Fortunately, in recent years, there has been increasing social attention for the development of family-like and small-scale forms of residence. These forms of residence offer a more relationship- and development-oriented climate than the present large living groups. Therewith they may better reflect the complex and heterogeneous problems which are characteristic of adolescents in SRYC.

### Unexpected finding

Prior to the start of the interviews, some concerns were raised by stakeholders about the capacity of adolescents to reflect on their own experiences of suicidality and self-harm and their ability to emotionally protect themselves from experiencing distress when discussing these sensitive issues. One possible explanation for these findings maybe the belief among professionals that incidents of suicide become higher when ideation and related behaviors are discussed [48]. Contrary to what stakeholders feared, all respondents indicated that rather than being harmed by their participation in the study, it was they experienced it as a relieving, positive experience. For some adolescents, the interview was the first time to discuss the subject of suicidality in depth.

“*I really liked talking so openly about it*. *I’ve never talked about being suicidal and stuff so extensively*, *really*. *It helps me as it relieves me to talk about it*.*” (D19*, *girl*, *age 17)*

Hence, these findings may help us understand the vital role the target group themselves can play in identifying factors that contribute to positive treatment outcomes concerning suicidal behavior in SRYC.

### Implications for practice

The findings of this study underline the importance of relational aspects in suicide prevention. Moreover, our findings show that fear and lack of knowledge regarding suicidal behavior (including self-harm) may influence group workers’ abilities to effectively help suicidal adolescents. This could be damaging to adolescent’s mental health, as residential workers may underestimate the iatrogenic effect of coercive interventions. We recommend that group workers are better supported in their contacts with adolescents in order to be able to manage this difficult work. More attention needs to be paid to the skills of group workers in residential institutions to develop and maintain positive relationships with adolescents and create a positive group climate. The support may include training, coaching, and supervision, and needs to be focused on specific situations, such as interactions with suicidal adolescents. Furthermore, the long-term impact of a completed suicide on adolescents and group workers in SRYC remains unclear. We recommend to conduct further research into these topics.

Although it is well established from a variety of studies, that group climate plays a critical role in residential youth care [77–79, 105] there is no literature available on the specific relationship between group climate and the prevalence of suicide. However, as cross sectional research data in other residential youth care fields do indicate causal relations between for example group climate and treatment motivation [77] and aggression [34] it can conceptually be assumed that group climate influences the prevalence of suicidal behavior in SRYC as based on the reports of adolescents in this study. Our suggestion for future research is to investigate this relationship in depth.

### Limitations, strengths and methodological considerations

Our findings should be interpreted with care. Firstly, the analysis is based on eleven interviews with adolescents residing in SRYC. Although this provided detailed subjective reports from which novel information could be drawn, the number of interviews does not permit generalization. Secondly, all adolescents were female, limiting the possibility to analyze gender differences. We recommend conducting further research on this subject on male adolescents. Thirdly, no information was available on forehand on the suicidal backgrounds or the numbers on suicidality in the general population in SRYC. Because of the importance of the subject, we decided not to differentiate between adolescents with NSSI and suicidal behavior. Subsequent studies should examine the specificities of each group in detail.

An unique feature of this study is the role of experience experts. We advocate that engaging experience experts and adolescents in research is a meaningful way to ensure best practice in suicide prevention, and to develop policies that meet the needs of adolescents in care systems [106, 107]. In line with observations by Holland (2009), putting the perspectives of youth and experienced experts central has resulted in interesting new findings [108]. This implies that including adolescents’ perspectives in research and policy may be promising for embracing changes and innovation in SRYC.

## Conclusion

This study contributes to the need for more specific knowledge on the subject of suicide prevention and suicidal behavior in living groups in SRYC. Our findings have two major implications for both research and policymaking. Firstly, adolescents prefer group workers who react responsively to suicidal behavior. Responsive, sensitive responses decreases adolescents’ suicidal distress. Secondly, the findings support the need to reduce the use of involuntary seclusion in response to suicidal behavior to zero. Overall, adolescents considered the care for suicidal adolescents in SRYC as inadequate. According to the interviewed adolescents the current SRYC system in the Netherlands does not do justice to the needs of suicidal young people.

## Supporting information

S1 File COREQ checklist. (DOCX)
